# Clinical characteristics of patients with COVID-19-associated mucormycosis (CAM) hospitalized at Shafa Hospital, Kerman: 2022

**DOI:** 10.1186/s12879-025-11861-y

**Published:** 2025-10-28

**Authors:** Ayeh Shamsadini, Fatemeh Fakhrabadi, Darya Shamsadini, Shiva Pouradeli

**Affiliations:** 1 Clinical Research Development Unit, Shafa Hospital, Kerman University of Medical Sciences, Kerman, Iran; 2 Isfahan University of Medical Sciences, Isfahan, Iran

**Keywords:** Mucormycosis, COVID-19, Black fungus, Hospital, Infection

## Abstract

**Background:**

Mucormycosis, commonly known as ‘black fungus,’ has emerged as a significant health threat during the COVID-19 pandemic, with a marked increase in prevalence among infected individuals. This study focuses on identifying the characteristics of COVID-19-associated mucormycosis patients in Kerman.

**Methods:**

This cross-sectional study was conducted at Shafa Hospital, Kerman, in 2022, following ethical approval from Kerman University of Medical Sciences. Patients with confirmed mucormycosis and positive COVID-19 PCR results were included using a census method. Diagnosis involved PCR testing, histopathological examination, and imaging studies. Data were collected from medical records by a trained student, ensuring anonymity. SPSS version 25 was used for analysis, with significance set at *p* < 0.05.

**Results:**

Among 37 patients with COVID-19-associated mucormycosis in 2022, 34 had complete medical records. The mean age was 58.8 ± 17.2 years, with a gender distribution of 58.8% female and 41.2% male. Mucormycosis was diagnosed 2–4 weeks after COVID-19 infection in 55.9% of cases. Pulmonary involvement was noted in 97.1% of patients, and corticosteroid use was prevalent in 85.3%. Diabetes and hypertension were present in 64.7% and 35.3% of patients, respectively. Opioid use was significantly higher in males (75.0%) compared to females (25.0%) (*P* = 0.042).

**Conclusions:**

This study reveals a higher prevalence of mucormycosis among older adults and individuals with underlying health conditions, particularly those who are immunocompromised, following COVID-19. The timing of diagnosis suggests a link between COVID-19-related immune compromise and mucormycosis onset, highlighting the need for vigilant monitoring during recovery. The significant pulmonary involvement underscores the necessity for routine respiratory screenings in at-risk populations. Additionally, the higher opioid use among males indicates a need to reassess pain management protocols to consider their impact on immune function. Understanding these patterns can inform targeted interventions to improve patient outcomes in diverse healthcare settings.

## Introduction

Mucormycosis, a life-threatening fungal infection commonly referred to as ‘black fungus,’ [1, 2] was previously regarded as an uncommon condition but has gained prominence amidst the COVID-19 pandemic [3], which began in late 2019 and caused widespread infection and significant mortality [4].

COVID-19-associated mucormycosis (CAM) has become a particularly serious concern during this time [5].A meta-analysis revealed a pooled prevalence of CAM at 7 cases per 1,000 COVID-19 patients, a rate approximately 50 times higher than the baseline prevalence of mucormycosis (0.14 cases per 1,000 patients) [6]. Factors contributing to this increased prevalence include diabetes, steroid use, and immunosuppression, conditions frequently observed among COVID-19 patients [1, 2, 7].

Common clinical manifestations of CAM include soft tissue swelling, necrosis, and black eschar on the palate, with maxillary bone and sinus involvement frequently observed [8]. The clinical presentation of CAM typically manifests within two to three weeks post-COVID-19 onset, with the sinuses being the most commonly affected area [9, 10].

Bacterial and fungal co-infections have been documented in patients with severe COVID-19, raising concerns about opportunistic fungal infections due to the immunocompromised state of these patients. CAM is particularly prevalent among those suffering from acute respiratory distress syndrome and those requiring prolonged stays in intensive care units (ICUs) [1, 11].

Research indicates that COVID-19 patients receiving high doses of corticosteroids, immunomodulators, and broad-spectrum antibiotics are particularly vulnerable to such infections [12–14]. Furthermore, studies show that hyperglycemia and excessive corticosteroid use can significantly worsen outcomes for COVID-19 patients [15, 16].

Concurrent infections can lead to longer hospital stays, an increased need for invasive procedures, and higher mortality rates [17]. Mucormycosis may emerge as a complication of COVID-19 in high-risk patients, underscoring the importance of vigilant monitoring by healthcare providers [12, 18, 19].

The prevalence of CAM has surged in developing countries during the pandemic, prompting urgent attention from healthcare providers [16, 20]. In regions with high COVID-19 cases, the prevalence of CAM has become a significant concern, with reports indicating a global prevalence ranging from 0.005 to 1.7 cases per million population. Notably, countries like India have reported exacerbated incidences due to the intersection of COVID-19, diabetes, and corticosteroid use [21–23].

This article aims to examine the demographic and clinical profiles of patients with CAM in Kerman. By doing so, we seek to identify individuals at risk for this disease in the region. This analysis is essential for developing effective prevention strategies, facilitating early diagnosis, and implementing comprehensive treatment plans. Our ultimate goal is to enhance patient outcomes and reduce the incidence of mucormycosis through targeted interventions.

## Methods

### Study design and ethical approval

This cross-sectional study was conducted in accordance with the Declaration of Helsinki, following ethical approval obtained from the Ethics Committee of Kerman University of Medical Sciences (IR.KMU.AH.REC.1401.059). The Ethics Committee waived the requirement for patient consent due to the retrospective and observational nature of the study. All patient data were anonymized prior to analysis to ensure privacy and confidentiality.

### Study population and location

The study population included all patients hospitalized at Shafa Hospital, Kerman in 2022.

### Inclusion criteria

Patients with confirmed mucormycosis were included in the study if they had positive COVID-19 PCR results. The census method was used to select patients from the ICUs.

### Diagnosis of COVID-19 and mucormycosis

COVID-19 was diagnosed using polymerase chain reaction (PCR) testing, performed on devices such as the QiAqunt965Plex Real-Time PCR system (QiAqunt965Plex Laboratories, Germany). Mucormycosis was diagnosed using the following criteria:

- Histopathology: Tissue invasion by fungal hyphae was confirmed using a Olympus CX31 microscope (Olympus CX31, Japan).
- Imaging: Radiological evaluations, including computed tomography (CT) and magnetic resonance imaging (MRI), were utilized to assess tissue and organ involvement consistent with mucormycosis.
- Clinical Judgment: In cases where histopathological was unavailable, diagnosis relied on clinical features and response to antifungal therapy.

### Data collection

Patient data were reviewed and extracted from medical files by a trained medical student using a standardized checklist. Information included demographic details (age, gender), underlying diseases, time since the onset of COVID-19 infection, history of corticosteroid, opium, and immunosuppressant usage, and the presence of pulmonary involvement related to COVID-19 infection. To ensure accuracy, laboratory test results were validated, and patients with incomplete or unclear information were excluded.

### Statistical analysis

Descriptive statistics were computed as frequency (%), mean ± standard deviation, and prevalence (95% CI). Clinical characteristics between genders were compared using the Chi-square test, and age differences were analyzed using the t-test. All statistical analyses were conducted using SPSS software version 25, with the significance level set at 0.05.

The formula for calculating the confidence interval (CI) for prevalence is:

$$\mathrm{CI}=\mathrm p\pm\mathrm Z\sqrt{\frac{\mathrm p\left(1-\mathrm p\right)}n}$$

Where:

- *p*: proportion $$\left(\mathrm p=\frac{\mathrm{cases}}{\mathrm{total}}\right)$$,
- *n*: total number of observations (*n* = 34),
- Z: Z-score for the desired confidence level (Z = 1.96 for 95%).

## Results

Out of 37 patients with CAM in 2022, 34 had complete medical files and were included in this study. The mean age of these patients was 58.8 ± 17.2 years (range: 25–90), with 58.8% (20 patients) being female and 41.2% (14 patients) being male. The mean age showed no significant difference between males and females (Table 1).

**Table 1:** Comparison of the average age of patients by gender

gender	*N*	Mean	Std. Deviation	*P*-value
Female	20	54.40	15.229	0.77
Male	14	65.00	18.489	

In most patients (55.9%), mucormycosis infection was diagnosed 2–4 weeks after being infected with COVID-19 (Table 2).

**Table 2:** Frequency of duration of COVID-19 infection in patients with CAM

Duration of COVID-19 infection	< 2 weeks	2–4 weeks	> 4 weeks
Frequency (%)	8 (23.5)	19 (55.9)	7 (20.6)

Pulmonary involvement was the most prevalent clinical characteristic among CAM patients, observed in 97.1% of cases (95% CI: 91.5–100%). Although its prevalence was higher in females (60.6%) than males (39.4%), the difference was not statistically significant (*P* = 0.412). Corticosteroid use was also highly prevalent, reported in 85.3% of patients (95% CI: 73.4–97.2%), with females accounting for 58.6% and males 41.4% (*P* = 1.000).

Diabetes was present in 64.7% of cases (95% CI: 48.9–80.5%), with a slightly higher prevalence in females (54.5%) compared to males (45.5%) (*P* = 0.493). Hypertension affected 35.3% of patients (95% CI: 19.6–51.0%), with a higher prevalence among females (58.3%) than males (41.7%), though the difference was not statistically significant (*P* = 0.966).

Immunosuppressant drug use was reported in 26.5% of patients (95% CI: 12.9–40.1%), with a slightly higher prevalence in males (55.6%) compared to females (44.4%) (*P* = 0.307). Opioid use was observed in 23.5% of cases (95% CI: 10.8–36.2%), significantly higher in males (75.0%) than females (25.0%) (*P* = 0.042).

Ischemic heart disease was documented in 17.6% of patients (95% CI: 4.7–30.5%), with no gender disparity (*P* = 0.672). Cancer and chronic kidney disease were the least prevalent conditions overall, each occurring in 11.8% of patients (95% CI: 0.8–22.8%), with equal distribution across genders (*P* = 1.000) (Table 3).

**Table 3:** Compared prevalence of clinical characteristics of CAM patients according by gender

Clinical Characteristics	Total Prevalence (%) [95% CI]	Female Prevalence (%) [95% CI]	Male Prevalence (%) [95% CI]	*P*-value
Pulmonary involvement	97.1 [91.5–100]	60.6 [43.9–77.3]	39.4 [22.7–56.1]	0.412
Corticosteroid consumption	85.3 [73.4–97.2]	58.6 [39.2–78.0]	41.4 [22.0–60.8]	1.000
Diabetes	64.7 [48.9–80.5]	54.5 [33.7–75.3]	45.5 [24.7–66.3]	0.493
Hypertension	35.3 [19.6–51.0]	58.3 [36.6–80.0]	41.7 [21.1–62.3]	0.966
Immunosuppressant drugs	26.5 [12.9–40.1]	44.4 [9.8–79.0]	55.6 [21.0–90.2]	0.307
Opioid consumption	23.5 [10.8–36.2]	25.0 [0.0–55.0]	75.0 [45.0–100.0]	0.042
Ischemic heart disease	17.6 [4.7–30.5]	50.0 [1.0–99.0]	50.0 [1.0–99.0]	0.672
Cancer	11.8 [0.8–22.8]	50.0 [1.0–99.0]	50.0 [1.0–99.0]	1.000
Chronic kidney disease	11.8 [0.8–22.8]	50.0 [1.0–99.0]	50.0 [1.0–99.0]	1.000

## Discussion

This study investigated the clinical and demographic characteristics of 34 CAM patients hospitalized at Shafa Hospital, Kerman, in 2022, providing valuable insights into risk factors, gender disparities, and disease patterns, while expanding on existing literature and uncovering novel associations worthy of further exploration.

### Sex and age distribution

The study revealed that among patients with CAM, the percentage of females (58.8%) was higher than that of males (41.2%). This finding aligns with a study by Fazeli et al. (2021) in Kermanshah, Iran, which reported a 58.33% prevalence of CAM in females [24]. However, this contrasts with multiple global studies reporting a male predominance in CAM cases, highlighting significant regional variations.

Globally, mucormycosis has shown a higher incidence in males, particularly among COVID-19 patients. For instance, Tavakolpour et al. (2022) reported that among 94 CAM cases in India, 63.5% were male and 36.5% were female, indicating a nearly 2:1 male-to-female ratio [25]. Similarly, Kumar et al. (2023) found a 67% male predominance in a multicenter study across India [26]. More strikingly, Singh et al. (2021) documented that 78.9% of CAM patients were male, while Pakdel et al. (2021) reported 66% male cases in Tehran [16, 18]. These differences suggest potential regional, genetic, or comorbidity-related influences on gender distribution.

Possible Explanations for Gender Disparity.

1. Immunological Differences: Emerging evidence suggests gender-based variations in immune responses to COVID-19 [27], which may influence susceptibility to secondary fungal infections.
2. Hormonal Factors: Further investigation is needed to explore how hormonal factors, including estrogen, may influence susceptibility to severe fungal infections [28].
3. Comorbidity Burden: Diabetes mellitus (DM), a major risk factor for mucormycosis, is more prevalent among males in many regions [29, 30]. Although, in this study, DM prevalence in females (54.5%) is slightly higher than in males (45.5%), potentially contributing to the observed female predominance in CAM cases.

The discrepancy in gender distribution across studies underscores the need for population-specific risk assessments. While some regions report female predominance (e.g., Iran), most studies from India and other high-burden countries indicate a male skew. Early diagnosis and aggressive management remain critical, particularly in high-risk groups such as diabetic and immunocompromised patients. Recognizing these variations can lead to tailored prevention and treatment strategies that consider gender differences in immune response and disease progression.

The mean age of CAM patients in this study was 58.8 ± 17.2 years, with males having a nonsignificantly higher mean age of 65 years. These results align with global data suggesting that mucormycosis disproportionately affects middle-aged to elderly individuals, particularly those with underlying comorbidities [16, 31]. A systematic review reported the median age of CAM patients as 53 years across diverse studies [32], while studies from India and Iran identified mean ages of 56.4 and 52 years, respectively, within ranges of 14 to 79 years [18, 31, 33]. While some studies indicate a slight male age predominance, these differences do not reach statistical significance [34, 35].

Our findings indicate that CAM primarily affects middle-aged to elderly individuals, consistent with global trends. However, the mean ages reported in various studies vary significantly. This highlights the need for targeted screening and early diagnosis protocols for older populations, especially in regions with a high prevalence of diabetes.

### Timing of mucormycosis diagnosis

Most patients were diagnosed with mucormycosis between two to four weeks after the onset of COVID-19 infection. In Iran, the average diagnosis occurred 25 days after COVID-19 onset [24], while Pakdel’s study reported an average of only seven days [18].

Globally, a systematic review found a median diagnosis time of 15 days for mucormycosis [36]. Another review indicated a median of 10 days (range 0–90 days) post-COVID-19 diagnosis [37]. Additionally, a systematic review and meta-analysis showed a median time of around 20 days between COVID-19 and mucormycosis diagnoses [38]. A study on rhino-orbital mucormycosis reported a mean interval of approximately 14 days [39], while studies in India indicated a median of 28 days (IQR, 15–45) after recovery from COVID-19 [40].

The variability in the timing of mucormycosis diagnosis across different regions highlights the need for improved awareness and monitoring protocols. Health systems should implement standardized guidelines for early identification and management of fungal infections in COVID-19 patients, particularly in settings where the incidence of CAM is rising.

### Prevalence of comorbidities

Similar to other studies, the most common underlying disease among CAM patients was diabetes, with a frequency of 64.7% [1]. Hypertension was prevalent in 35.3% of patients with CAM.

In Eshraghi’s study, the prevalence of diabetes in CAM patients was 82.8% [41], while in Baral’s study, diabetes was nearly 80%, and hypertension was more than 38% of CAM cases [28]. In India, diabetes was present in 71.4% and hypertension in 29.8% of CAM cases [42].). In Tavakolpour’s study, the prevalence of diabetes in CAM cases was 55.8%, and hypertension was 21.2% [25]. The high prevalence of diabetes among CAM patients is likely due to the immunocompromised state resulting from uncontrolled blood sugar levels [43]. Maintaining optimal glycemic control and closely monitoring patients with diabetes or other immunocompromising conditions is essential to reduce the risk of mucormycosis in COVID-19 patients.

The prevalence of hypertension in CAM patients is a significant concern, as hypertension is a common comorbidity in COVID-19 patients and may contribute to the development of mucormycosis. Studies indicate that hypertension is prevalent among CAM patients, with varying degrees of severity and outcomes [44, 45]. In the context of mucormycosis, hypertension is recognized as a notable risk factor, although its prevalence among these patients is reported less frequently compared to other conditions, such as diabetes. The interplay of these comorbidities underscores the complexity of managing COVID-19 patients with multiple health issues.

The high prevalence of diabetes and hypertension among CAM patients reinforces the need for integrated management approaches for individuals with these comorbidities. Public health policies should focus on optimizing glycemic control and hypertension management, especially in the context of COVID-19 recovery. This can help reduce the risk of opportunistic infections like mucormycosis.

### Pulmonary involvement

In this study, 97.1% of CAM patients exhibited pulmonary involvement, consistent with the aggressive nature of mucormycosis. Another study, which included 101 cases of CAM, reported pulmonary involvement in 8 cases [16]. A systematic review by Özbek found a prevalence of underlying pulmonary disease at 5%, revealing a significant mortality rate of 56.1% in patients with pulmonary disease, compared to 37.4% in those without this comorbidity [32]. This stark contrast highlights the impact of pulmonary involvement on outcomes and reinforces the need for targeted interventions in managing these complications.

The absence of significant gender differences in pulmonary involvement indicates that this complication is common across both sexes. This finding advocates for a uniform approach to diagnosing and treating pulmonary mucormycosis, regardless of gender, to mitigate risks and enhance patient survival rates.

### Corticosteroid and immunosuppressant use

A striking 85.3% of patients reported corticosteroid use, a known risk factor for the development of mucormycosis. Corticosteroid use among CAM patients was reported as 46.6% in Pakdel’s study, 75% in Fazeli’s study, and 76% and 61.2% in Singh and Ravi’s studies, respectively [16, 18, 24, 46].

Although corticosteroid consumption showed no significant gender difference, the high overall prevalence raises concerns about the judicious use of corticosteroids in COVID-19 patients, especially those with pre-existing conditions. While corticosteroids can significantly control inflammation caused by COVID-19 and improve respiratory conditions, excessive immune suppression may predispose patients to opportunistic infections, including mucormycosis.

Conversely, 26.5% of patients used immunosuppressant drugs, with no significant gender difference. The frequency of immunosuppressive drugs was 13% in Pakdel’s study [18]. The correlation between immunosuppressive therapy and increased susceptibility to opportunistic infections warrants further investigation.

### Opioid consumption

Notably, 23.5% of patients reported opioid use, revealing a significant gender disparity favoring males. In Kerman, the prevalence of opium consumption is 8.1% among the adult population, surpassing average rates observed in the country and Southwestern Asia. This prevalence is significantly higher in men than in women, reinforcing the notion that gender plays a crucial role in substance use patterns [47].

Opioids may indirectly contribute to CAM by affecting immune function. Chronic opioid use has been associated with immunosuppression, potentially increasing susceptibility to opportunistic infections like mucormycosis. Additionally, opioid use in ICU settings often coincides with other risk factors such as prolonged hospitalization and invasive procedures, further predisposing patients to CAM [48].

Furthermore, high opium consumption raises questions about pain management practices in CAM patients and highlights the need for tailored approaches to address gender-specific needs in pain management. It is essential to explore how these substance use patterns affect treatment adherence and recovery outcomes. Future studies should investigate the relationship between substance use, pain management practices, and health outcomes in both genders to develop effective, gender-sensitive interventions.

### Implications for global health strategies

Understanding these regional differences in CAM prevalence and characteristics can inform better management practices and public health policies in several ways:

- Tailored Interventions: Public health initiatives should be adapted to local demographics and comorbidity patterns. For instance, in regions with a higher female prevalence of CAM, educational campaigns could focus on risk factors specific to women, such as hormonal influences and diabetes management.
- Resource Allocation: Health authorities can allocate resources more effectively by identifying high-risk populations based on age, gender, and comorbidity profiles. This targeted approach can enhance early intervention strategies and improve patient outcomes.
- Collaborative Research: Encouraging international collaboration and data sharing among researchers can help build a comprehensive understanding of CAM. Global databases could facilitate the identification of trends and risk factors, leading to more effective prevention and treatment strategies.
- Policy Development: Policymakers should prioritize the establishment of guidelines for the management of COVID-19 patients at risk for mucormycosis. This includes developing protocols for the judicious use of corticosteroids and immunosuppressants, as well as monitoring for fungal infections during recovery.

## Conclusion

This study highlights that mucormycosis was more prevalent among older adults and individuals with underlying health conditions, particularly those who are immunocompromised, following COVID-19. The timing of mucormycosis diagnosis following COVID-19 indicates a potential link between immune compromise associated with COVID-19 and the development of mucormycosis. The significant prevalence of pulmonary involvement suggests that healthcare providers should conduct routine screenings for respiratory symptoms in at-risk populations to enable early detection and intervention. Moreover, the significantly higher opioid use among males may reflect gender-specific patterns in pain management or substance use, necessitating a reassessment of pain management protocols that consider the implications of opioid use on immune function.

Recognizing regional variations in the characteristics of CAM patients is vital for shaping effective global health strategies. This knowledge can guide the development of targeted interventions, such as enhanced screening protocols and tailored treatment plans, ultimately improving management practices and patient outcomes in diverse healthcare settings.

### Limitation and suggestion

The retrospective nature of the study may limit the ability to establish causality, which should be considered when interpreting the results. Additionally, the relatively small sample size could affect the generalizability of the findings. However, this study was conducted during a specific and challenging period when cases of COVID-19-associated mucormycosis were emerging. Due to the unique conditions at that time, this disease was very rare, and all the cases recorded during one year of the study were collected. Therefore, our aim was to provide early insights into this critical issue, which can serve as a foundation for larger studies in the future.

Reporting these cases in our area, considering the rarity of the disease, can help enhance our understanding of its prevalence. Furthermore, our study contributes to the growing body of literature on COVID-19 complications, particularly mucormycosis, by focusing on a specific population in Kerman.

Moreover, the notable gender differences in opioid use and their implications for immune function represent a unique aspect of our findings.

To gain deeper insights into the characteristics of CAM patients, conducting a comparative study with other hospitalized patients in ICUs is recommended.

Furthermore, future research should focus on the long-term outcomes of these patients and the impact of different treatment regimens on their prognosis.

Although data regarding the type of oxygenation methods, length of stay in the ICUs, specific antibiotics used, and immunosuppressive agents administered were not collected in this study, these factors could further elucidate the clinical course of CAM and should be considered in future research.

## Data Availability

The datasets analyzed during the current study are not publicly available due to privacy concerns but are available from the corresponding author on reasonable request.
